# First Results for the Elemental Composition of Copaiba Oil Resin (*Copaífera Spp.*) from Flona Carajás By ICP–OES

**DOI:** 10.1021/acsomega.5c08791

**Published:** 2025-12-22

**Authors:** Charles M. S. Borges, Patricia de O. Nunes, Jonathan B. O. Souza, Isamara de S. C. Benathar, Selma L. Goulart, Fábio I. M. Carvalho, Marcos Rodrigues, Heronides A. D. Filho, Kelly G. F. Dantas

**Affiliations:** † Institute of Exact and Natural Sciences, 37871 Federal University of Pará—UFPA, Rua Augusto Corrêa, 01, Bairro Guamá, Belém, Pará 66075-110, Brasil; ‡ 89116 Federal Rural University of the Amazon (UFRA), Avenida Duane Silva Souza S/N, PA 275, Zona Rural, Parauapebas, Pará 66077-530, Brasil; § Federal Rural University of the Amazon (UFRA), Avenida Presidente Tancredo Neves, N° 2501, Terra Firme, Belém, Pará 66077-830, Brasil

## Abstract

Copaiba oil resin is a rich source of bioactive compounds, and its extraction not only generates income for local communities but also contributes to forest conservation and the regional bioeconomy. In this study, the inorganic composition of copaiba oil resin obtained in the Carajás National Forest (FLONA Carajás), in the Brazilian Amazon, was determined by inductively coupled plasma optical emission spectrometry (ICP–OES). Ten samples were digested in a microwave oven using 5 mL of HNO_3_ (7 mol L^–1^) and 2 mL of H_2_O_2_ (30% m/m) and analyzed using ICP–OES. The elements Al, As, Ca, Cd, Cr, Cu, Fe, K, Mg, Na, Ni, Pb, Sb, Se, Ti, and Zn were quantified, with concentrations ranging from 0.055 mg kg^–1^ (Cd) to 2355.9 mg kg^–1^ (Ca). Recoveries obtained by the analyte addition method ranged from 87.2% to 108.7%, indicating good precision and minimal matrix effects. The Shapiro–Wilk test indicated that the data did not follow a normal distribution, and Spearman’s correlation analysis revealed significant positive correlations (*r* = 0.872–0.999) between darker-colored samples and higher concentrations of specific elements. The method proved efficient for quantifying the inorganic composition of copaiba oil resin. These results provide the first report of the inorganic profile of copaiba oil resin from FLONA Carajás and highlight the importance of elemental characterization to support the sustainable extraction and valorization of this nontimber forest product from the Amazon.

## Graphical abstract

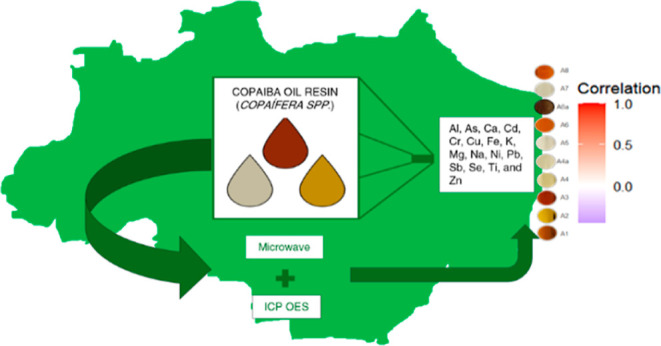

## Introduction

The importance of the Amazon Rainforest in regulating the global climate and its great biodiversity place the region at the center of the bioeconomy debate, thus highlighting the need to create a bioeconomy for the Amazon that values economic viability but guarantees sustainability and environmental protection^1,2^

Nontimber forest products are goods that emphasize the maintenance of ecosystem services provided by trees and, in a broader sense, by forests. Despite their growing recognition as viable economic alternatives to traditional agricultural and timber production in many tropical forests, several challenges stand in the way of their wider adoption, including the lack of standardized production processes that meet the stringent requirements of more demanding markets^1^

In the southeast of the Pará state, there is the Carajás National Forest (Flona Carajás), a conservation unit for sustainable use in the Brazilian Amazon, whose main activity is mining. This forest has the potential for the growth of bioeconomy-related activities, including the extraction of copaiba oil resin; however, studies on such activities in the region are still incipient^3^

Copaibeira is a large tree from which copaiba oil resin is extracted and is considered a rich source of active compounds.^4,5^ Copaiba oil resin can be used pure (fresh or distilled) by oral administration or topical application, where it is used as a healing, anti-inflammatory, antiseptic, and antitumor agent and as an agent to treat bronchitis and skin diseases. In addition, it can be consumed as a component of products such as ointments, soaps, and syrups^5,6^ )

The use of various vegetables and other natural products, such as copaiba oil resin, in herbal medicine has become widespread in recent years. Therefore, there is growing interest in studies on their chemical compositions, both for organic constituents, which have their primary medicinal effects, and inorganic constituents such as macronutrients, micronutrients, and toxic elements.^7^ The composition of inorganic elements in copaiba oil resin has not yet been reported. There are existing studies on the organic chemical composition of copaiba oil resin, where its anti-inflammatory,^8^ antibacterial,^9^ photochemical,^10^ and volatile components^11^ are evaluated.

As the quality of oils is directly related to the concentration of trace metals, multielement analysis has gained more importance in recent years. This type of analysis can be considered innovative and useful for obtaining nutritional and toxicity information, which is critical for its commercialization.^12^ However, there are still a few studies on multielemental determination in Amazonian nonwood products such as copaiba oil resin.

Multielement analysis of oil samples is particularly difficult because some elements are present at very low levels. Sample preparation is a critical step in the entire analytical procedure, making the analysis extremely susceptible to contamination during preparation and requiring sensitive instrumental methods. In addition, its high viscosity makes it difficult to introduce the sample into the equipment, and its high organic load increases the matrix effect and the possibility of polyatomic molecular interferences from elements such as C, N, and S. This high organic content can result in carbon deposition in the sampling cone and loss of sensitivity.^12–16^

Analyzing oil samples is more challenging because of the complexity of the matrix. Pretreatment is a crucial step in the multielement analysis of oils, and microwave-assisted digestion is the most commonly used sample preparation method for determining metals in oils and other complex organic matrices.^17,18^ Microwave radiation has been described as a successful assistant for sample pretreatment in analytical chemistry, where a closed system is used to avoid volatile compound losses and reduce the number of reagents required for sample preparation and the hypothesis of sample contamination.^14^

Atomic spectrometric methods are popular for the determination of trace elements in vegetable and biodiesel samples.^19,20^ Metals in vegetable oils are typically determined using atomic spectrometric techniques such as flame absorption spectrometry (FAAS),^21^ graphite furnace atomic absorption spectrometry (GFAAS),^22^ inductively coupled plasma optical emission spectrometry (ICP–OES),^23^ inductively coupled plasma mass spectrometry (ICP–MS),^24^ and microwave-induced plasma optical emission spectrometry (MIP–OES).^15,25^

Inductively coupled plasma optical emission spectroscopy (ICP–OES) or atomic emission spectroscopy (ICP–AES) is a widely used analytical technique for the multielement analysis of a wide range of samples. The operating conditions of ICP–OES can be optimized by following simple procedures, and it is relatively easy to use. The advantages of the ICP–OES technique include its multielement capability for around 75 elements, acceptable sensitivities and limits of detection (LOD), and its ability to analyze samples of diverse matrices, such as agricultural, environmental, geochemical, metallurgical, petrochemical, and worn metals.^26^ The literature reports studies using ICP–OES for multielement determination in various types of matrices, such as fish,^27^ soil,^28^ geopropolis,^29^ and petroleum samples.^30^

However, no studies have characterized the inorganic constituents of copaiba oil resin, in addition to it being a complex and highly viscous Amazonian matrix. The microwave digestion method and ICP–OES determination are an alternative to overcome challenges such as matrix effects and the high organic load present in natural oils and resins. The determination of efficient methods can contribute both to the prospecting potential of this extractive activity and to the possibility that this oil resin obtained from the Carajás National Forest has a chemical composition different from those in other locations in the Amazon due to the characteristics of the region’s soil,^31^ with a predominant area of mining exploration. Therefore, this study aimed to determine Al, As, Ca, Cd, Cr, Cu, Fe, K, Mg, Na, Ni, Pb, Sb, Se, Ti, and Zn in copaiba oil resin samples obtained from FLONA Carajás by ICP–OES.

## Results and Discussion

### Accuracy Assessment

The evaluation of the accuracy of the ICP–OES analysis procedure presented in Table 1 showed good results for most of the elements. As, Ca, Cd, Cr, Cu, Mg, Na, Ni, Pb, Sb, Ti, and Zn achieved recoveries between 87.2% and 108.7%. The recovery values found were within the acceptable range of 80%–120%^31^ and showed that the analyses by ICP–OES were performed without any matrix effects or spectral interference.

**Table 1:** Shows the Recovery Values Obtained for the Elements Investigated Using the Analyte Addition and Recovery Method

	concentrations found in mg kg^–1^ and their recoveries		
elements	1.5	2.5	3.5
Al	1.40 (93.29%)	2.11 (84.57%)	3.13 (89.50%)
As	1.50 (100.00%)	2.35 (93.87%)	3.15 (90.00%)
Ca	1.31 (87.19%)	2.74 (109.78%)	3.07 (87.65%)
Cd	1.47 (98.53%)	2.26 (90.40%)	3.24 (92.58%)
Cr	1.44 (95.77%)	2.23 (89.14%)	3.22 (92.06%)
Cu	1.43 (95.12%)	2.27 (91.77%)	3.40 (97.23%)
Fe	1.41 (94.16%)	2.25 (89.88%)	3.23 (92.28%)
K	1.63 (108.74%)	2.28 (91.22%)	3.57 (101.92%)
Mg	1.42 (94.45%)	2.24 (89.74%)	3.25 (92.89%)
Na	1.35 (90.08%)	2.32 (92.80%)	3.31 (94.46%)
Ni	1.55 (103.22%)	2.41 (96.33%)	3.23 (92.11%)
Pb	1.50 (99.98%)	2.33 (93.51%)	3.23 (92.36%)
Sb	1.53 (102.31%)	2.29 (91.7%)	3.18 (90.90%)
Se	1.54 (102.84%)	2.35 (94.15%)	3.23 (92.30%)
Ti	1.44 (95.91%)	2.27 (90.84%)	4.22 (117.52%)
Zn	1.49 (99.51%)	2.37 (94.76%)	3.35 (95.80%)

The evaluation of the efficiency of the sample preparation procedure presented good recoveries for all elements investigated, where the values obtained were within the acceptable recovery range of 80%–120%.^31^ Only Fe showed recoveries outside this range, with recoveries between 130.4% and 134.23% (Table 2). These recoveries may be due to an error in the addition of Fe before digestion.

**Table 2:** Assessment of the Accuracy of the Sample Preparation Procedure Using the Analyte Addition and Recovery Method

	concentrations found in mg kg^–1^ and their recoveries		
elements	1.5	2.5	3.5
Al	1.50 (100.25%)	2.42 (97.04%)	3.32 (94.97%)
As	1.44 (96.26%)	2.37 (94.84%)	3.38 (96.67%)
Ca	1.58 (105.64%)	2.38 (95.46%)	3.58 (102.30%)
Cd	1.53 (102.18%)	2.43 (97.25%)	3.48 (99.43%)
Cr	1.63 (108.96%)	2.62 (105.05%)	3.78 (107.95%)
Cu	1.70 (113.73%)	2.74 (109.83%)	3.86 (110.27%)
Fe	2.01 (134.26%)	3.26 (130.36%)	4.60 (131.52%)
K	1.60 (107.32%)	2.54 (101.46%)	3.66 (104.67%)
Mg	1.46 (97.18%)	2.40 (96.00%)	3.30 (94.27%)
Na	1.48 (98.96%)	2.47 (98.95%)	3.35 (95.87%)
Ni	1.50 (100.60%)	2.41 (96.52%)	3.38 (96.54%)
Pb	1.47 (98.13%)	2.43 (97.23%)	3.48 (99.44%)
Sb	1.46 (97.32%)	2.40 (96.10%)	3.37 (96.41%)
Se	1.61 (107.26%)	2.64 (105.48%)	3.85 (109.95%)
Ti	1.64 (109.13%)	2.69 (107.67%)	3.79 (108.41%)
Zn	1.32 (88.38%)	2.16 (86.32%)	3.07 (87.32%)

### Figures of Merits

The LODs for Al, As, Ca, Cd, Cr, Cu, Fe, K, Mg, Na, Ni, Pb, Sb, Se, Ti, and Zn are exhibited in Table 3.

**Table 3:** SBR, Reference Concentration (*C*_0_), BEC, LOD, LOQ, and *R* ^2^ Obtained in the Determination of Analytes in Oil Resin by ICP–OES

element	SBR	*C*_0_ (mg kg^–1^)	BEC (mg L^–1^)	LOD (mg kg^–1^)	LOQ (mg kg^–1^)	*R* ^2^
Al	79.82	5	0.0626	0.32	1.08	0.999
As	11248	5	0.0004	0.003	0.01	0.999
Ca	23.10	5	0.2164	0.47	1.56	0.994
Cd	5906.8	5	0.0009	0.002	0.01	0.999
Cr	397.68	5	0.0126	0.12	0.41	0.999
Cu	324.83	5	0.0154	0.09	0.30	0.999
Fe	328.60	5	0.0152	0.21	0.71	0.998
K	9.04	5	0.5531	1.48	4.93	0.999
Mg	875.12	5	0.0057	0.01	0.03	0.999
Na	129.58	5	0.0386	0.09	0.31	0.999
Ni	3294.90	5	0.0015	0.003	0.01	0.999
Pb	859.17	5	0.0058	0.04	0.13	0.999
Sb	2288.10	5	0.0022	0.02	0.06	0.999
Se	1413.20	5	0.0035	0.02	0.08	0.999
Ti	1079.70	5	0.0049	0.02	0.05	0.999
Zn	387.11	5	0.0129	0.001	0.004	0.999

Studies on these analytes in copaiba oil resin have not been reported. However, the LODs obtained in this study were better than those reported by^32^ in the analysis of edible oils with acid digestion in a microwave oven and determined by inductively coupled plasma optical emission spectrometry (ICP–OES). These LODs found are also lower than those reported by^33^ for elements in pumpkin seed oil using acid digestion and ICP–OES, and those reported by^34^ who analyzed argan oil using ICP–OES. The LOD values are shown in Table 4.

**Table 4:** LOD Values Found in the Literature for Edible Oils

references	elements	LOD (mg kg^–1^)
^32^	ICP–OES	1.04 (Al), 0.08 (Ca), 0.05 (Cd), 0.81(Cr), 0.32 (Cu), 0.41 (Fe), 0.06 (Mg), 0.26 (Ni), 0.22 (Pb), 0.07 (Ti), and 0.09 (Zn)
^33^	ICP–OES	0.92 (Al), 0.04 (Cd), 0.88 (Cr), 0.29 (Cu), 0.32 (Fe), 0.03 (Mg), 0.14 (Na), 0.16 (Ni), 0.17 (Pb), 0.04 (Ti), and 0.05 (Zn)
^34^	ICP–OES	0.35 (As), 11 (Ca), 0.03 (Cd), 0.12 (Fe), 9 (K), 3 (Mg), 2.5 (Na), 0.05 (Ni), 0.2 (Pb), 0.8 (Se), 0.03 (Ti), and 0.04 (Zn)

### Quantification of Analytes by ICP–OES

Table 5 shows the concentrations of Al, As, Ca, Cd, Cr, Cu, Cu, Fe, K, Mg, Na, Ni, Pb, Sb, Se, Ti, and Zn in copaiba oil resin by ICP–OES after microwave acid digestion in an oven with a cavity. The results for the investigated elements are expressed in mg kg^–1^. The precision of the procedure was evaluated by triplicate determinations of each sample, and the relative standard deviation (RSD) values were below 10% for most elements, indicating good repeatability of the ICP–OES measurements.

**Table 5:** Element Concentrations (mg kg^–1^) in Copaiba Oil Resin by ICP–OES and Their Respective Standard Deviations (*n* = *3*)

elements	A1	A2	A3	A4	A4a	A5	A6	A6a	A7	A8
Al	<0.32	7.20 ± 0.04	25.55 ± 1.26	11.96 ± 0.68	19.93 ± 1.68	3.18 ± 0.04	10.51 ± 0.54	10.80 ± 1.61	<0.32	14.62 ± 0.26
As	<0.003	<0.003	0.17 ± 0.01	<0.003	<0.003	<0.003	<0.003	<0.003	<0.003	<0.003
Ca	639.59 ± 35.87	451.53 ± 3.17	2355.91 ± 143.13	<0.47	<0.47	<0.47	140.80 ± 1.80	105.20 ± 98.50	99.44 ± 24.53	1324.92 ± 41.80
Cd	0.06 ± 0.01	<0.002	<0.002	<0.002	<0.002	<0.002	<0.002	<0.002	<0.002	<0.002
Cr	2.00 ± 0.17	1.40 ± 0.25	<0.12	3.81 ± 0.44	<0.12	<0.12	0.60 ± 0.08	2.04 ± 0.20	<0.12	2.18 ± 0.13
Cu	9.88 ± 0.23	10.89 ± 1.18	14.06 ± 1.26	7.72 ± 0.46	5.32 ± 0.63	<0.09	8.50 ± 0.50	5.07 ± 0.15	0.30 ± 0.03	5.49 ± 0.82
Fe	16.75 ± 1.58	4.70 ± 0.15	29.41 ± 0.42	3.63 ± 0.28	<0.21	<0.21	9.80 ± 0.50	<0.21	<0.21	3.91 ± 0.19
K	<1.48	<1.48	153.95 ± 4.32	<1.48	<1.48	<1.48	<1.48	<1.48	<1.48	<1.48
Mg	21.68 ± 0.21	4.90 ± 0.17	34.76 ± 0.11	2.64 ± 0.29	5.86 ± 1.92	6.05 ± 2.56	3.25 ± 0.01	39.22 ± 25.93	<0.01	20.16 ± 1.02
Na	80.94 ± 8.30	63.82 ± 2.03	94.94 ± 4.77	60.66 ± 1.35	20.83 ± 2.16	<0.09	26.90 ± 0.12	47.65 ± 6.01	13.64 ± 0.59	43.90 ± 0.18
Ni	<0.003	<0.003	<0.003	0.01 ± 0.001	1.12 ± 0.07	0.04 ± 0.002	0.02 ± 0.002	1.43 ± 0.36	1.31 ± 0.11	3.50 ± 0.49
Pb	<0.04	1.05 ± 0.04	<0.04	0.16 ± 0.01	1.15 ± 0.11	0.84 ± 0.01	0.35 ± 0.01	0.63 ± 0.07	<0.04	0.24 ± 0.02
Sb	<0.02	<0.02	<0.02	<0.02	<0.02	<0.02	<0.02	<0.02	<0.02	<0.02
Se	0.51 ± 0.06	0.35 ± 0.01	0.56 ± 0.02	0.68 ± 0.04	0.63 ± 0.04	1.04 ± 0.07	0.52 ± 0.03	<0.02	0.84 ± 0.02	0.67 ± 0.08
Ti	<0.02	<0.02	<0.02	<0.02	<0.02	<0.02	<0.02	<0.02	<0.02	<0.02
Zn	6.53 ± 0.18	2.30 ± 0.75	3.64 ± 0.32	2.72 ± 0.64	0.61 ± 0.47	<0.001	2.07 ± 0.02	7.76 ± 1.36	2.54 ± 0.38	17.60 ± 0.93

Among the elements analyzed in the copaiba oil resin samples, the values for Sb and Ti were below the LOD, those for As and K were above the LOD only for sample A3 (0.17 ± 0.01 and 153.95 ± 4.32 mg kg^–1^), and that for Cd was above the LOD only for sample A1 (0.055 ± 0.005 mg kg^–1^). No existing studies on the levels of inorganic constituents in copaiba oil resin were found to allow for a comparison with our results. However, some studies have evaluated the inorganic constitution of other vegetable oils (Table 6). Comparing the results found for K in copaiba oil resin with those found in other studies, our values were higher than those found by^35^ (11.0 ± 0.12 mg kg^–1^) in babassu oil and below those found by^36^ (397.6 ± 16.48 mg kg^–1^) in andiroba oil.

**Table 6:** Determination of Inorganic Constituents in Vegetable Oils

references	sample	preparation	analysis	results
^35^	babassu oil (*Attalea speciosa*)	microwave acid digestion	ICP–OES	24.35 ± 2.780 (Na); 1.10 ± 0.012 (K); 4.10 ± 0.06 (Ca); 2.25 ± 0.014 (Mg); 0.13 ± 0.002 (Fe); 0.36 ± 0.011 (Cr); 0.09 ± 0.008 (Se); 1.03 ± 0.003 (Al); 0.45 ± 0.054 (Zn); and <LD (Ni, Cu, Co, Cd) mg 100 g^–1^
 ^37^	sunflower oil (*Helianthus annuus* L.)	microwave acid digestion	ICP–OES	12 ± 0.4 (Ca); 2.7 ± 0.11 (Cu); 8.6 ± 0.21 (Fe); 23.5 ± 0.4 (K); 25.1 ± 0.5 (Mg); 12.4 ± 0.4 (Na); 2.2 ± 0.12 (Zn); <LD (Al, Co, Cr, Ni, Pb, Ti) mg kg^–1^
 ^36^	andiroba oil	microwave acid digestion	MIP–OES	5.3 ± 0.42 (Al), 273.9 ± 2.58 (Ca), 397.6 ± 16.48 (K), 3.2 ± 0.16 (Mg), 7.7 ± 0.23 (Pb), 8.8 ± 0.78 (Si), 13.9 ± 1.25 (V), 2.1 ± 0.078 (Zn), 152.4 ± 11.4 (Na), <LD (B, Ba, Cd, Co, Cr, Cu, Fe, Li, Mn, Ni) mg kg^–1^
 ^15^	olive oil (*Olea europaea* L.), evening primrose oil (*Oenothera biennis*), avocado oil (*Persea americana*), coconut oil (*Cocos nucifera*)	microwave acid digestion	MIP–OES	0.06 ± 0.01–0.12 ± 0.02 (Al); 0.014 ± 0.006–0.070 ± 0.003 (Cr); 0.92 ± 0.02–1.7 ± 0.1 (Ni); <LD (<0.007) – 0.04 ± 0.01 (Ti); <LD (<0.0013) – 0.12 ± 0.01 (Zn) mg kg^–1^

Aluminum is considered a neurotoxic agent that can increase the likelihood of developing Alzheimer’s disease, as well as cause cognitive impairment and neurological diseases. Aluminum can interfere with certain essential elements, such as calcium, and calcium metabolism is one of the most important processes in the human body.^38^ Al was found in most samples at levels between 3.18 ± 0.04 and 25.55 ± 1.26 mg kg^–1^. These values were close to those found by^35^ (10.3 ± 0.03 mg kg^–1^) and^36^ (5.3 ± 0.42 mg kg^–1^) and above those found by^15^ (0.06 ± 0.01–0.12 ± 0.02 mg kg^–1^) in different vegetable oils. Samples A1 and A7 had values below the LOD.

The Ca values found in the samples varied from 99.44 ± 24.53 to 2355.9 ± 143.13 mg kg^–1^, which is close to that found by^36^ (273.9 ± 2.58 mg kg^–1^) for some samples but above the concentrations reported by^35^ (41.0 ± 0.6 mg kg^–1^) and^37^ (12 ± 0.4 mg kg^–1^) in sunflower oil samples. The Ca levels in samples A4, A5, and A4a were below the LOD.

Copper is a trace element that has several biochemical functions in living organisms. It plays an important role in cardiac function, osteogenesis, carbohydrate metabolism, and collagen tissue lipogenesis. It strengthens the immune system. It also has an effect on plant growth. Excessive consumption has a toxic effect on the body, preventing the performance of some enzymes. Fe is a necessary and useful element for organisms and a significant part of tissue and blood in animal and human bodies. It enters the structure of the hemoglobin that forms erythrocytes and has an important role in the formation of blood and function.^39^ For Cu, only sample A5 obtained a value below the LOD. All other samples exhibited levels between 0.300 ± 0.026 and 14.06 ± 1.26 mg kg^–1^. For Fe, levels between 3.63 ± 0.28 and 29.41 ± 0.42 mg kg^–1^ were found in 6 of the 10 samples; only the values for samples A5, A7, A4a, and A6a were below the LOD. The levels of Cu and Fe are higher than those reported by^35^ (Fe = 1.3 ± 0.02 mg kg^–1^; Cu < LOD) and close to those found in sunflower oil by^37^ (Cu = 2.7 ± 0.11 mg kg^–1^; Fe = 8.6 ± 0.21 mg kg^–1^).

Chromium in its hexavalent form possesses high mobility in soil, permeability through biological membranes, and the capacity to generate reactive oxygen species, thereby disrupting DNA integrity and protein function.^40^ The Cr levels in the samples were between 0.60 ± 0.08 and 3.81 ± 0.44 mg kg^–1^, with the values for samples A3, A5, A7, and A4a falling below the LOD. The levels of Ni in the samples ranged between 0.011 ± 0.0008 and 3.50 ± 0.49 mg kg^–1^, except for A1, A2, and A3, whose values were below the LOD. The Ni concentration levels in the copaiba oil resin samples were close to those reported by^15^ (0.92–1.7 mg kg^–1^) and^35^ (3.6 ± 0.11 mg kg^–1^)

Magnesium is a cofactor of more than 300 enzymatic reactions, acting either on the enzyme itself as a structural or catalytic component or on the substrate, especially for reactions involving ATP, which makes magnesium essential in the intermediary metabolism for the synthesis of carbohydrates, lipids, nucleic acids, and proteins. Magnesium deficiency can cause hipocalcemia and hypokalemia, leading to neurological or cardiac symptoms when it is associated with marked hypomagnesemia.^41^ The concentrations of Mg ranged from 2.64 ± 0.29 to 39.22 ± 25.93 mg kg^–1^, with only the value for sample A7 being below the LOD. The Zn concentrations in the samples were between 0.61 ± 0.47 and 17.6 ± 0.93 mg kg^–1^. Only in the A5 sample was the Zn concentration below the LOD. These results were close to those reported by^37^ (Mg = 25.1 ± 0.5 mg kg^–1^; Zn = 2.2 ± 0.12 mg kg^–1^),^35^ (Mg = 22.5 ± 0.14 mg kg^–1^; Zn = 4.5 ± 0.54 mg kg^–1^), and^36^ (Mg = 3.2 ± 0.16 mg kg^–1^; Zn = 2.1 ± 0.078 mg kg^–1^).

Na^+^ is the dominant cation in the extracellular fluid (ECF) of the body. The functions of sodium lie in its participation in the control of the volume and systemic distribution of total body water; enabling the cellular uptake of solutes. Sodium chloride added during industrial food processing and discretionary use or food preservation is the major source of dietary sodium in Western diets. Other sources of sodium include inherently native sources and sodium-containing food additives, in which sodium may be associated with anions other than chloride.^42^ The levels of Na were between 13.64 ± 0.59 and 94.94 ± 4.77 mg kg^–1^, except for sample A5, which presented a lower LOD. These values were lower than those reported by^36^ (152.4 ± 11.4 mg kg^–1^) and^35^ (243.5 ± 27.80 mg kg^–1^) and close to those reported by^37^ (12.4 ± 0.4 mg kg^–1^).

Pb can be found in edible vegetable oils as a result of environmental contamination, refining processes, transfer during transport, or packaging processes. In addition, Pb is a heavy metal that accumulates in the body, altering cellular metabolism and causing various harmful effects on human health, such as a decrease in the number of erythrocytes needed for the synthesis of red blood cells and hemoglobin, due to the inhibition of enzymes caused by exposure to this heavy metal.^43^ The levels of Pb in the samples varied between 0.16 ± 0.01 and 1.15 ± 0.11 mg kg^–1^, and only the values for samples A1, A3, and A7 were below the LOD. These concentrations were lower than those reported by^36^ (7.7 ± 0.23 mg kg^–1^).

Selenium was once considered a toxic element, but today it is recognized that selenium is an essential element necessary for various biological functions in humans and animals. Selenium is critical for optimal human health due to its antioxidant activity. Selenium protects the body from oxidative stress, reducing cellular damage caused by free radicals.^44^ The Se concentrations in the copaiba oil resin samples were between 0.35 ± 0.01 and 1.04 ± 0.07 mg kg^–1^, except for sample A6a, whose value was below the LOD. The concentrations are close to those found by^35^ (0.9 ± 0.08 mg kg^–1^).

The difference between the levels of the chemical elements found in the copaiba oil resin samples is probably due to the fact that they were collected from different trees and at different collection sites. Even in trees in nearby locations, the elemental concentrations within the tree can fluctuate. Several factors can influence the concentration of chemical elements in copaiba oil resin, such as the type of copaiba species, soil, topography, altitude, tree health, absorption, and transport of chemical elements contained in the soil absorbed by the root to other parts of the tree.^45^

### Statistical Analysis

As shown in Table 7, Pearson correlation coefficient analysis of the copaiba oil resin samples exhibited a significant positive correlation between samples A1, A2, A3, A6, A6a, and A8, with an *r* value between 0.627 and 0.954. Samples A4, A4a, A5, and A7 showed no significant correlation with any of the other samples.

**Table 7:** Pearson Correlation for Copaiba Oil Resin Samples^a^

	A1	A2	A3	A4	A4a	A5	A6	A6a	A7	A8
A1	1									
A2	0.800**	1								
A3	0.900**	0.891**	1							
A4	0.345	0.564	0.427	1						
A4a	–0.009	0.318	0.227	0.336	1					
A5	0.036	0.091	0.273	–0.064	0.236	1				
A6	0.782**	0.954**	0.927**	0.591	0.200	0.145	1			
A6a	0.627*	0.800**	0.709**	0.327	0.391	0.027	0.700*	1		
A7	0.254	0.309	0.291	0.027	0	–0.454	0.382	0.391	1	
A8	0.782**	0.827**	0.854**	0.336	0.291	–0.045	0.800**	0.909**	0.500	1

a ** = *p* < 0.5; * = *p* < 0.1.

With the correlation values between the samples, it is possible to see a relationship between the content of the elements and the color of the samples (Figure 1). Samples A1, A2, A3, A6, A6a, and A8 are darker in tone and have the highest concentration values for some elements. For example, sample A3 has the highest concentrations of Al, Ca, Cu, Fe, and Na, which may be related to its shade, being the darkest among the samples. This pattern was also repeated in other samples. Samples A4, A4a, A5, and A7 have a less intense color and the lowest contents for some elements; they show a weak correlation with all the other samples that have a more intense color tone.

**Figure 1:**
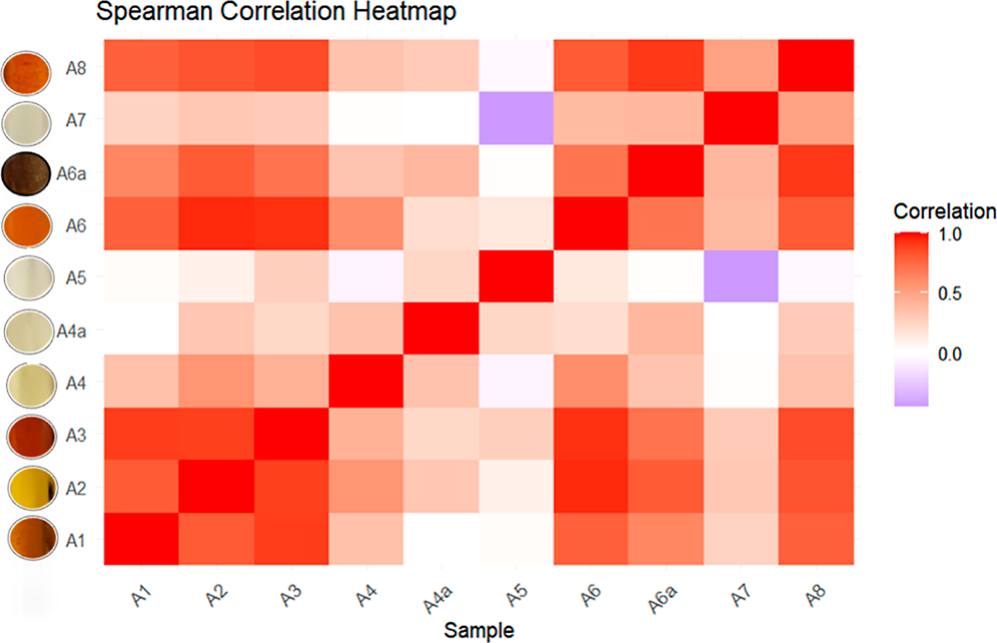
Heatmap of the Spearman correlation coefficients results of the copaiba oil resin samples.

The results obtained in this study reveal a consistent and distinct elemental profile for copaiba oil resin samples from the Carajás National Forest, highlighting the influence of the collection site and tree-specific factors on the inorganic composition. These findings not only provide the first comprehensive report of the inorganic composition of copaiba oil resin from this region but also establish an analytical baseline that may guide future investigations on the relationship between the chemical, physicochemical, and biological properties of Amazonian nontimber forest products.

## Conclusions

This study presents the first investigation into the elemental composition of copaiba oil resin. The protocol proposed in this study, with the acid digestion of the samples with nitric acid and peroxide, and the determination of the elements by ICP–OES, proved effective for quantifying the examined elements. Except for Sb and Ti, all the elements studied were found in at least one sample, with a wide variation in their levels. The analysis of Pearson correlation coefficients presented a relationship between samples with a more intense color tone and how this characteristic may be linked to the content of some of the elements studied. A significant positive correlation was observed between samples obtained from the same tree. The analytical procedure established for ICP–OES, applied to a complex organic matrix of copaiba oil resin, represents an innovative analytical application for natural products from the Amazon. As a limitation, the restricted availability of samples and natural variability among trees may influence the representativeness of the results, but the methodology proved to be robust and reliable for the intended purpose. The inorganic composition results of this study can be further applied to investigate the organic composition and other physicochemical properties of copaiba oil resin and contribute to the valorization of this product and the activity of extracting copaiba oil resin, which is a source of income for many Amazonian families and communities and plays an important role in preserving biodiversity and conserving the Amazon rainforest.

## Experimental Section

### Samples

We collected samples from *Copaifera* spp. individuals in Carajás National Forest, located in southern Pará, in the Brazilian Amazon (Figure 2). The geographical position of individuals was recorded with the GPS. For oil resin extraction, we perforated the trunk at a height of 1.3 m above the soil surface using a metal auger (diameter of 1.587 cm) adapted to a treadmill with a length of 48 cm. A polyvinyl chloride pipe coupled to a plastic container was introduced into the hole to collect the oil resin. After the flow ended, the hole was sealed to avoid contact of the tree with living agents (fungi, microbes), which could compromise tree health.

**Figure 2:**
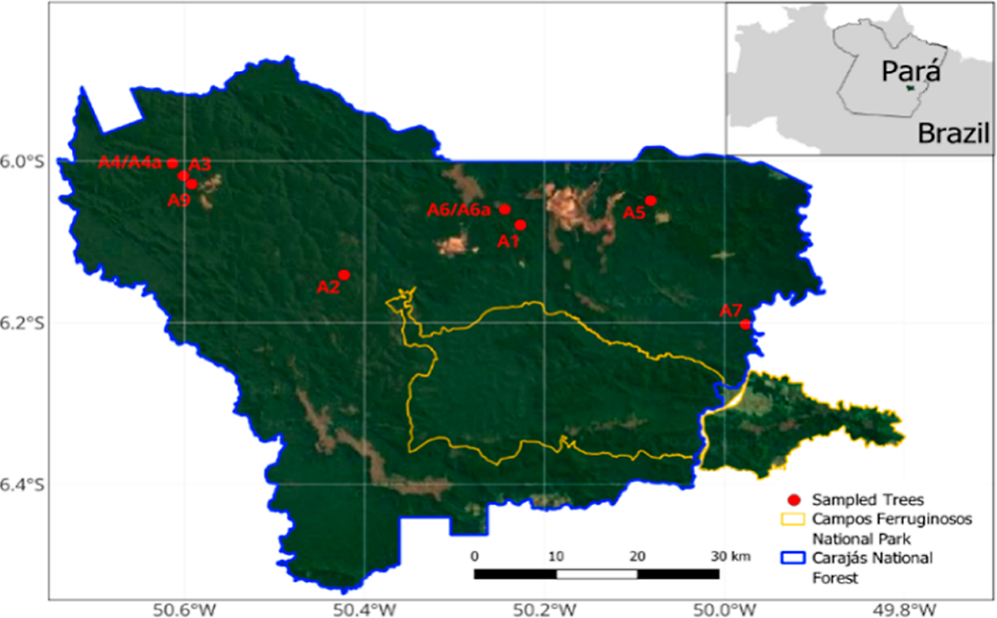
Map of the FLONA Carajás region and its respective sample collection points.

The first extraction occurred in October 2018, where 50 *Copaifera* spp. individuals were perforated to collect oil resin; however, only 8 individuals produced sufficient oil resin for multielement analysis (samples A1, A2, A3, A4, A5, A6, A7, and A8). In November 2019, we performed a second extraction of oil resin from these eight individuals; however, only two individuals produced oil resin (named samples A4a and A6a). The yield and absence of oil resin in Copaifera trees have been reported in the literature.^3,46^ Figure 3 shows the oil resin samples.

**Figure 3:**
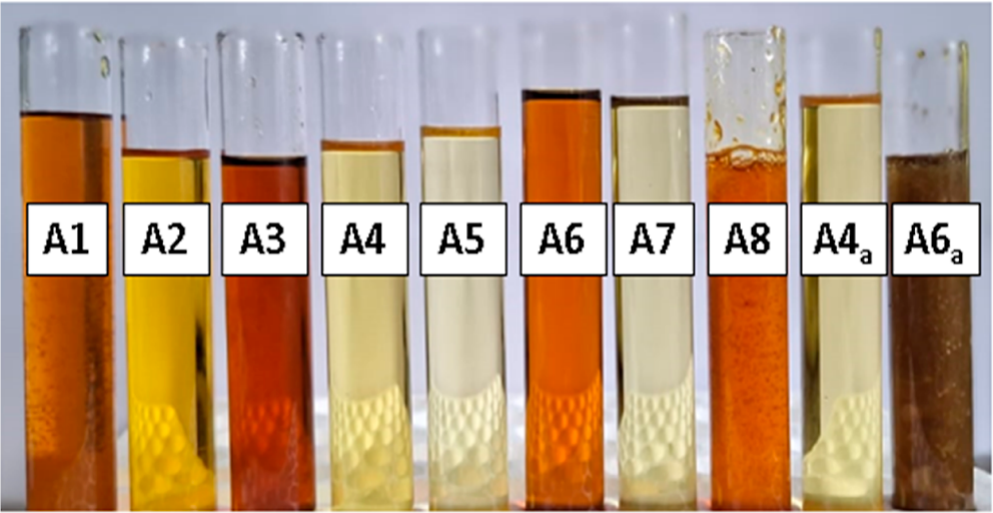
Samples of copaiba oil resin from the FLONA Carajás region.

### Reagents and Solutions

All reagents used were of analytical grade. All solutions were prepared using ultrapure water with a resistivity of 18.2 MΩ cm obtained using a Synergy-UV purification system (Millipore, Bedford, USA).

All solutions and samples were stored in previously decontaminated polyethylene bottles.

Nitric acid (65% v v^–1^; Sigma-Aldrich, USA), previously purified using a subdistillation system (Berghof, model BSP 929-IV, Germany), and hydrogen peroxide (30% m/m; Neon Comercial, Suzano, SP) were used for acid digestion.

Standard solutions for preparing the calibration curve were prepared in HNO_3_ 5% v v^–1^ medium, from the appropriate dilution of the stock solution containing 1000 mg L^–1^ of Al, As, Ca, Cd, Cr, Cu, Fe, K, Mg, Na, Ni, Pb, Sb, Se, Ti, and Zn (Sigma-Aldrich, USA).

### Instrumentation

Acid digestion of the samples was performed in a microwave oven with a cavity (Start E model, Milestone, Sorisole, Italy).

To determine the elements Al, As, Ca, Cd, Cr, Cu, Fe, K, Mg, Na, Ni, Pb, Sb, Se, Ti, and Zn in the copaiba oil resin samples, an ICP–OES system (iCAP 6500 Duo Model, Thermo Fisher Scientific, Cambridge, England) with two configurations (axial and radial) and iTEVA operating software was used. The digested samples were introduced into the plasma using a concentric nebulizer and a cyclonic nebulizer chamber. The operating parameters followed those recommended by the manufacturer, which were as follows: 1.15 W of radio frequency, 12 L min^–1^ of plasma gas flow, 0.5 L min^–1^ of auxiliary gas flow, and 0.5 L min^–1^ of gas flow in the nebulizer. All wavelengths were chosen to obtain the highest emission peak and the absence of spectral interference with the signal of the other elements, with the following lines atomic (I) or ionic (II): Al I: 396.152 nm; As I: 189.042 nm; Ca II: 396.847 nm; Cd I: 228.802 nm; Cr II: 283.563 nm; Cu I: 327.396 nm; Fe I: 238.204 nm; K I: 769.896 nm; Mg II: 279.553 nm; Na I: 589.592 nm; Ni II: 221.647 nm; Pb I: 216.999 nm; Sb I: 206.833 nm; Se I: 196.090 nm; Ti II: 336.121 nm; Zn II: 202.548 nm. All measurements were performed using argon (99.999% purity, White Martins, Belém, PA, Brazil) to purge the optics and form the plasma.

### Digestion Procedure

The digestion procedure was performed according to the protocol recommended by the manufacturer of the microwave digestion system (Milestone, model Start E) for oil and organic matrices, with minor adjustments to sample mass and reagent volumes. During the digestion procedure, 0.25 g of each copaiba oil resin sample (*n* = *3*) was weighed directly into the microwave digestion bottles, followed by the addition of an oxidizing mixture containing 5 mL of HNO_3_ (7 mol L^–1^), 2 mL of H_2_O_2_ m/m, and 1 mL of ultrapure water. The vials were inserted into a microwave oven with a cavity, and the samples were digested using a procedure involving three steps: 800 W, 180 °C for 15 min (ramp); 800 W, 180 °C for 15 min (step); and 50 min of ventilation. After digestion, the solution was filtered using a quantitative filter paper, and its volume was adjusted to 20 mL with ultrapure water. To determine the levels of Al, As, Ca, Cd, Cr, Cu, Fe, K, Mg, Na, Ni, Pb, Sb, Se, Ti, and Zn, an aliquot of the digests was removed and diluted to 10 mL, obtaining a final acidity of 5%. Analytes were determined by ICP–OES.

## Procedure for Assessing Accuracy

The ICP–OES analysis procedure was evaluated using the analyte addition and recovery method. Aliquots of 1.5, 2.5, and 3.5 mg L^–1^ for each element investigated were added to those digested and then analyzed by ICP–OES.

Similar to the analysis procedure, the efficiency of the sample preparation step was evaluated by adding aliquots of 1.5, 2.5, and 3.5 mg L^–1^ of the analytes to the samples before digestion and subsequent analysis by ICP–OES.

The limit of detection (LOD) and limit of quantification (LOQ) were calculated using background equivalent concentrations (BECs), signal-to-background ratios (SBRs), and relative standard deviation (RSD b) of the intensities of ten consecutive measurements of the blank solution, according to the following equations: LOD = (3 RSD b × BEC)/100 and LOQ = (10 RSD b × BEC)/100.^47^

### Statistical Analysis

All statistical analyses and tests were performed using RStudio 2024.12.0 Build 467 “Kousa Dogwood.” The normality of the experimental data was assessed using the Shapiro–Wilk test, where most elemental concentration data sets had *p*-values <0.05, indicating that they did not follow a normal distribution. In addition, the limited sample size (*n* = 10) indicates that nonparametric statistical methods are considered more appropriate. Spearman’s correlation coefficient was applied to assess the relationships between variables, as this method does not assume data normality and is robust for nonparametric data sets.
